# Risk factors and prevention strategies for shoulder injuries in overhead sports: an updated systematic review

**DOI:** 10.1186/s40634-022-00493-9

**Published:** 2022-08-16

**Authors:** Matthias Wilhelm Hoppe, Joana Brochhagen, Thomas Tischer, Knut Beitzel, Romain Seil, Casper Grim

**Affiliations:** 1 Movement and Training Science, Leipzig University, Jahnallee 59, 04109 Leipzig, Germany; 2 Clinic for Orthopaedics and Trauma Surgery, Malteser Waldkrankenhaus St. Marien, Erlangen, Germany; 3 Department of Orthopaedics, University Medical Centre Rostock, Rostock, Germany; 4 ATOS Orthoparc Klinik, Cologne, Germany; 5 Department of Orthopaedic Surgery, Centre Hospitalier Luxembourg and Luxembourg Institute of Health, Luxembourg, Luxembourg; 6 Centre for Musculoskeletal Surgery Osnabrück (OZMC), Klinikum Osnabrück, Osnabrück, Germany; 7 Institute for Health Research and Education (IGB), Osnabrück University, Osnabrück, Germany

**Keywords:** Baseball, Handball, Joint instability, Long biceps tendinosis, Overuse injuries, Rehabilitation, Return to sports, Rotator cuff lesion, Shoulder pain, Tennis

## Abstract

**Purpose:**

The aim of this systematic review was to update the knowledge on risk factors and prevention strategies for shoulder injuries in overhead sports with special emphasis on methodological quality.

**Methods:**

All methodological procedures were performed in line with a previous systematic review by Asker et al. (2018). The literature search was conducted in the PubMed, Google Scholar, Cochrane, and SPORT-Discuss databases. Due to the risk of bias assessment, only studies with at least an acceptable methodological quality were included. A best-evidence synthesis was performed to clarify the evidence and direction of the risk factors and prevention strategies.

**Results:**

A total of nine studies were included in the data extraction process. One study had a high and eight studies had an acceptable methodological quality. Seven cohort studies investigated risk factors and two randomised controlled trails evaluated prevention strategies. Moderate evidence was found for two non-modifiable (playing position, gender) and three modifiable factors (shoulder rotational strength, scapular dyskinesia, shoulder prevention programme) that were associated with the shoulder injury risk. All further risk factors had moderate and no association with risk (shoulder rotational ROM, joint position sense) or limited (history of shoulder/elbow pain, age, training experience, training volume, school grade, playing level), and conflicting evidence (setting).

**Conclusions:**

There is moderate evidence for two non-modifiable (playing position, gender) and three modifiable factors (shoulder rotational strength, scapular dyskinesia, shoulder prevention programme) being associated with the shoulder injury risk in overhead sports.

**Supplementary Information:**

The online version contains supplementary material available at 10.1186/s40634-022-00493-9.

## Introduction

Shoulder pain is one of the most common musculoskeletal complaints and can be extremely debilitating [47] for athletes in overhead sports [41]. In these sports, the shoulder joint is at high risk for overuse injuries due to their similar load and risk profiles [10, 15, 27, 38]. They all have repetitive and explosive overhead movements in common that could lead – in case of overload – to an ongoing process of tissue damage [1, 33]. The incidence and prevalence of shoulder injuries in overhead sports varies from 0.2/1000 to 1.8/1000 hours [7, 31, 50] and from 5% to 36% [9, 10, 32], respectively. The time loss from sport-specific training can range between four to 6 months [29], whereas the return-to-sports rates vary between 20% and 90% [29, 46]. In professional baseball, as one of the most shoulder demanding overhead sport, return-to-performance rates of 7% have been reported for some injuries [16]. Overall, the burden of shoulder injuries in overhead sports can be severe, potentially career-threatening, and therefore underlines the need to develop appropriate prevention strategies for both the athletes’ health and long-term performance development.

For the development of prevention strategies, knowledge of the epidemiology and aetiology as well as risk factors are important [17, 49]. For overhead sports, clinically established modifiable risk factors are: insufficient load management, abnormal throwing or stroking technique, previous injury to the upper extremity and/or spine, functioning of the kinetic chain, deficits in shoulder range of motion (ROM) or strength, scapular dyskinesia, and posture as “slough-position”. Essential non-modifiable risk factors are: male sex, young age, individual anatomy as torsion of the humerus or glenoid dysplasia, and high capsular laxity [8, 11, 13, 18, 19]. However, compared to other severe sport injuries, especially to the anterior cruciate ligament where meta-analysis of meta-analysis exist [52], there is clearly less evidence on risk factors and prevention strategies for shoulder injuries [3, 24], and thus more research is needed.

In 2018, a comprehensive review on risk factors and prevention strategies for shoulder injuries in overhead sports was published [3]. From 4776 identified studies, 17 studies on risk factors and one study on prevention strategies fulfilled the inclusion criteria and were considered for data extraction. However, no study with a high methodological quality could be included. Since many studies on risk factors and prevention strategies for shoulder injuries have been published during the last 3 years, an update is required. Thereby, and to allow valid practical recommendation, it is rational to place a focus on studies with, at least in part, an acceptable methodological quality.

The aim of this systematic review was to update the knowledge on risk factors and prevention strategies for shoulder injuries in overhead sports with special emphasis on methodological quality.

## Methods

### Research design

The Preferred Reporting Items for Systematic Reviews and Meta-analyses statement (PRISMA) was followed [25]. To provide an update, all methodological procedures were performed in line with a previous systematic review on risk factors and prevention strategies for shoulder injuries [3]. Briefly, our eligibility criteria were: (i) randomised controlled trials or cohort/case-control studies published in English; (ii) more than 20 athletes per group of any gender, age, and playing level; (iii) badminton, baseball, cricket, handball, lacrosse, softball, tennis, volleyball, and water polo as overhead sports; and (iv) shoulder injury or pain as dependent outcome variable. All methodological steps were conducted by two authors and a third made a decision on disagreements. Due to the non-invasive character, no ethical approval was considered.

### Literature search strategy and study selection

The literature search was conducted in the PubMed, Google Scholar, Cochrane, and SPORT-Discuss databases. Subsequent to the previous review [3], the publication period was restricted from 15 May 2017 to 31 December 2020. The applied search terms were taken from the previous review and combined by Boolean operators. The received entries were downloaded to a reference manager (Endnote X9). All reference lists of the included studies were screened for additional studies fulfilling the eligibility criteria. After duplicates were removed, the abstracts and full texts of the remaining studies were checked for their fit by taking the eligibility criteria into account.

### Risk of bias assessment

The risk of bias assessment was performed using the Scottish Intercollegiate Guidelines Network (SIGN) checklists [42] in a modified version developed by the previous review [3]. Thereon, the internal validity of all studies was evaluated based on 15 and 10 items for cohort/case-control studies and randomised controlled trails, respectively. According to the SIGN-guidelines, the overall assessment of each study was stated as: “high quality”, “acceptable”, “borderline”, and “unacceptable”. The criteria of these ratings are described in detail elsewhere [3]. The risk of bias assessment for those studies published before 15 May 2017 were taken from the previous review [3].

### Data extraction

Contrary to the previous review [3], only studies with a high or an acceptable methodological quality rating were included in the data extraction process. Additionally, studies with at least an acceptable methodological quality from the previous review were included. The reason was that we aimed to provide valid practical recommendations for which, at least in part, an acceptable methodological quality is an essential prerequisite. The data extraction of the studies was conducted according to the PICO-framework [30]. An additional meta-analysis was not conducted due to the large heterogeneity of the studies. Instead, and according to the previous review [3], a best-evidence synthesis was performed to clarify the evidence and direction of the risk factors and prevention strategies. In Table 1, the corresponding criteria are defined and the ratings were as follows: “strong evidence”, “moderate evidence”, “limited evidence”, “conflicting evidence”, and “no evidence”.

**Table 1:** Criteria for the best-evidence synthesis

Rating	Study quality	Criterion
Strong evidence	≥ 2 high quality studies	≥ 75% consistent findings in these studies
Moderate evidence	1 high quality study and/or ≥ 2 moderate quality studies	≥ 75% consistent findings in these studies
Limited evidence	1 moderate quality study and/or ≥ 1 low quality studies	n/a
Conflicting evidence	≥ 2 studies of any quality	< 75% consistent findings in these studies
No evidence	No admissible studies were found	

## Results

### Literature search strategy, study selection, and risk of bias

**Fig. 1:**
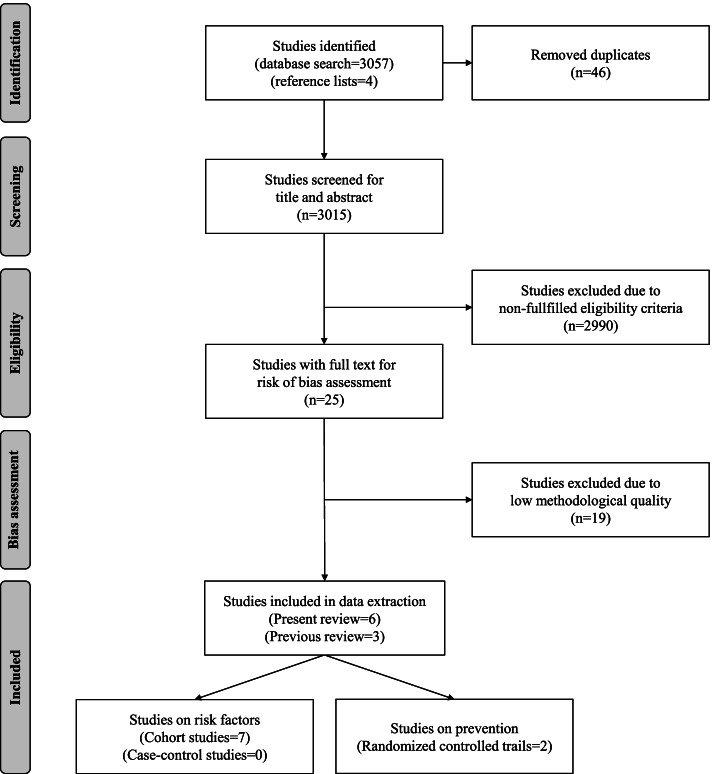
Flow chart of the literature search strategy according to the PRISMA-guidelines

Figure 1 shows the results of the literature search strategy, including the outcomes of the study selection and risk of bias assessment procedures. Of the initial 3057 studies found, 25 complied with the initial inclusion criteria and were assessed for risk of bias assessment. Table 2 summarises the corresponding outcomes by the SIGN-checklists. Due to a low methodological quality (borderline and unacceptable ratings), 19 studies were excluded (for references see supplementary material). Thus, 6 studies with high and acceptable methodological qualities from our [4, 5, 20, 35, 39, 40] and 3 studies with acceptable ratings from the previous review [2, 26, 54] were included in the data extraction process. Of the 9 included studies, one study had an overall high quality [5] and 8 studies an acceptable risk of bias rating [2, 4, 20, 26, 35, 39, 40, 54].

**Table 2:** Outcomes of the 25 studies checked for the risk of bias assessment by the SIGN-checklist

Study	Item															Total				Overall assessment
	1.1	1.2	1.3	1.4	1.5	1.6	1.7	1.8	1.9	1.10	1.11	1.12	1.13	1.14	1.15	Y	N	NA	CS	
Asker et al. (2020) [5]	Y	Y	Y	Y	4%	N	Y	N	N	Y	Y	Y	Y	Y	Y	11	3	0	0	High quality
Andersson et al. (2016) [2]^a, b^	Y	Y	Y	N	Y	Y	Y	Y	CS	CS	–	–	–	–	–	7	1	0	2	Acceptable
Asker et al. (2018) [4]	Y	Y	Y	Y	6%	N	Y	NA	NA	Y	Y	Y	Y	Y	Y	11	1	2	0	Acceptable
Hams et al. (2019a) [20]	Y	Y	N	NA	15%	NA	Y	NA	NA	Y	Y	Y	Y	Y	Y	9	1	4	0	Acceptable
Matsuura et al. (2017) [26]^a^	Y	Y	Y	CS	12%	N	Y	NA	NA	N	N	N	Y	Y	Y	7	4	2	1	Acceptable
Oliver et al. (2019) [35]	Y	Y	N	NA	CS	NA	Y	N	N	Y	Y	Y	CS	Y	Y	8	3	2	2	Acceptable
Sakata et al. (2019) [39]^b^	Y	Y	Y	N	Y	Y	Y	7%/8%	CS	Y	–	–	–	–	–	7	1	0	1	Acceptable
Saper et al. (2018) [40]	Y	Y	N	NA	CS	NA	Y	N	N	Y	Y	Y	Y	Y	Y	9	3	2	1	Acceptable
Wilk et al. (2015) [54]^a^	Y	Y	Y	Y	CS	N	Y	Y	NA	Y	N	Y	CS	Y	Y	10	2	1	2	Acceptable
Achenbach et al. (2020)^c^	Y	Y	Y	CS	CS	CS	Y	Y	NA	Y	Y	Y	Y	Y	Y	11	0	1	3	Borderline
Agel et al. (2007)^a,c^	Y	Y	Y	Y	CS	N	Y	NA	NA	Y	N	NA	N	Y	NA	7	3	4	1	Borderline
Bonza et al. (2009)^a,c^	Y	Y	N	Y	CS	N	Y	NA	NA	Y	N	NA	N	Y	Y	7	4	3	1	Borderline
Cunado-Gonzales et al. (2019)^c^	Y	Y	Y	CS	29%	NA	Y	CS	CS	Y	Y	N	Y	N	Y	8	2	1	3	Borderline
Dick et al. (2007a)^a,c^	Y	Y	Y	Y	CS	N	Y	NA	NA	Y	N	NA	N	Y	NA	7	3	4	1	Borderline
Dick et al. (2007b)^a,c^	Y	Y	Y	Y	CS	N	Y	NA	NA	Y	N	NA	N	Y	NA	7	3	4	1	Borderline
Fares et al. (2020)^c^	Y	Y	NA	NA	NA	NA	Y	NA	NA	Y	NA	NA	Y	Y	Y	7	0	8	0	Borderline
Gardner et al. (2016)^a,c^	Y	Y	Y	Y	CS	N	Y	NA	NA	Y	N	NA	N	Y	Y	8	3	3	1	Borderline
Giroto et al. (2015)^a,c^	Y	Y	Y	Y	0%	NA	Y	NA	NA	Y	N	NA	N	Y	NA	7	2	5	0	Borderline
Hibberd et al. (2016)^a,c^	Y	Y	Y	Y	CS	N	Y	NA	NA	Y	N	NA	N	Y	Y	8	3	3	1	Borderline
Hinton et al. (2005)^a,c^	Y	Y	N	Y	CS	N	Y	NA	NA	Y	Y	NA	N	Y	Y	8	3	3	1	Borderline
Kim et al. (2020)^c^	Y	Y	Y	N	44%	N	Y	CS	N	Y	Y	N	Y	Y	Y	9	4	0	1	Borderline
Marshall et al. (2007)^a,c^	Y	Y	Y	N	CS	N	Y	NA	NA	Y	N	NA	N	Y	NA	6	4	4	1	Borderline
Møller et al. (2017)^a,c^	Y	Y	CS	Y	CS	N	Y	CS	N	Y	Y	Y	N	Y	Y	9	3	0	3	Borderline
Noonan et al. (2016)^a,c^	Y	Y	N	Y	CS	N	Y	Y	NA	Y	N	Y	N	N	Y	8	5	1	1	Borderline
Owens et al. (2009)^a,c^	Y	Y	Y	Y	CS	N	Y	NA	NA	Y	N	NA	N	Y	NA	7	3	4	1	Borderline
Reeser et al. (2015)^a,c^	Y	NA	N	Y	CS	N	Y	NA	NA	Y	N	NA	N	Y	NA	5	4	5	1	Borderline
Robinson et al. (2013)^a,c^	Y	Y	Y	Y	CS	N	Y	NA	NA	Y	N	NA	N	Y	Y	8	3	3	1	Borderline
Sallis et al. (2001)^a,c^	Y	Y	Y	Y	CS	N	Y	NA	NA	Y	N	NA	N	Y	Y	8	3	3	1	Borderline
Sekiguchi et al. (2020)^c^	Y	N	Y	N	16%	N	Y	N	N	CS	Y	Y	Y	Y	Y	8	5	0	1	Borderline
Takagishi et al. (2019)^c^	Y	Y	N	CS	0%	NA	Y	CS	N	Y	Y	N	Y	Y	Y	8	3	1	2	Borderline
Berardi et al. (2019)^c^	Y	Y	N	CS	CS	NA	Y	NA	NA	Y	Y	NA	CS	N	Y	6	2	4	3	Unacceptable
Bere et al. (2015)^a,c^	Y	Y	Y	Y	3%	NA	Y	NA	NA	Y	N	NA	N	N	NA	6	3	5	0	Unacceptable
Byram et al. (2010)^a,c^	Y	Y	Y	N	CS	N	Y	CS	N	Y	N	N	N	N	N	5	8	0	2	Unacceptable
Clarsen et al. (2014) [9]^a^	Y	Y	Y	N	> 20%	Y	Y	NA	NA	Y	Y	N	Y	Y	Y	10	2	2	0	Unacceptable
Dakic et al. (2018)^c^	Y	Y	Y	CS	0%	NA	Y	NA	NA	Y	Y	N	Y	Y	Y	9	1	3	1	Unacceptable
Dutton et al. (2019)^c^	Y	CS	Y	Y	CS	CS	Y	CS	N	Y	Y	N	Y	Y	Y	9	2	0	4	Unacceptable
Erickson et al. (2019)^c^	Y	Y	N	CS	CS	CS	Y	N	CS	Y	Y	CS	Y	N	Y	7	3	0	5	Unacceptable
Forthomme et al. (2013)^a,c^	Y	CS	N	Y	CS	N	Y	Y	NA	CS	N	Y	N	N	N	5	6	1	3	Unacceptable
Gregory et al. (2002)^a,c^	Y	N	Y	Y	CS	N	N	CS	N	Y	N	N	N	Y	N	5	8	0	2	Unacceptable
Hams et al. (2019b)^c^	Y	Y	N	Y	CS	CS	Y	N	N	Y	Y	CS	Y	Y	Y	9	3	0	3	Unacceptable
Hansen et al. (2019)^c^	Y	Y	NA	CS	CS	NA	Y	NA	NA	CS	Y	N	N	N	Y	5	3	4	3	Unacceptable
Luig et al. (2020)^c^	Y	N	NA	CS	0%	NA	Y	NA	NA	Y	Y	NA	Y	Y	Y	7	1	5	1	Unacceptable
Lyman at al. (2002)^a,c^	Y	Y	Y	CS	CS	N	N	CS	N	CS	N	Y	N	N	Y	5	6	0	4	Unacceptable
Lyman et al. (2001)^a,c^	Y	Y	Y	CS	CS	N	N	CS	N	CS	N	Y	Y	Y	Y	7	4	0	4	Unacceptable
Marchena-Rodriguez et al. (2020)^c^	Y	N	N	N	0%	NA	Y	Y	NA	Y	CS	N	N	Y	Y	6	5	2	1	Unacceptable
Oliver et al. (2018)^c^	Y	Y	Y	N	9%	N	Y	N	N	CS	Y	N	N	Y	Y	7	6	0	1	Unacceptable
Polster et al. (2013)^a,c^	Y	CS	N	NA	CS	N	Y	Y	NA	Y	N	N	N	Y	Y	6	5	2	2	Unacceptable
Polster et al. (2016)^a,c^	N	CS	N	N	CS	N	N	CS	N	Y	N	Y	N	Y	Y	4	8	0	3	Unacceptable
Ranson et al. (2008) [38]^a^	Y	CS	Y	Y	CS	N	Y	NA	NA	CS	N	N	N	N	Y	5	5	2	3	Unacceptable
Rugg et al. (2019)^c^	Y	N	NA	Y	NA	NA	Y	NA	NA	N	N	CS	Y	N	Y	5	4	5	1	Unacceptable
Salzer et al. (2020)^c^	N	N	N	CS	CS	NA	Y	N	N	CS	N	N	Y	N	Y	3	8	1	3	Unacceptable
Seil et al. (1998)^a,c^	Y	Y	N	N	CS	N	Y	NA	NA	Y	N	N	N	N	Y	5	7	2	1	Unacceptable
Sekiguchi et al. (2018)^c^	Y	N	Y	N	5%	N	Y	CS	N	CS	Y	N	N	Y	Y	6	6	0	2	Unacceptable
Sell et al. (2014)^a,c^	Y	N	Y	Y	CS	N	Y	NA	NA	CS	CS	NA	N	Y	Y	6	3	3	3	Unacceptable
Shanley et al. (2011)^a,c^	Y	Y	Y	N	CS	N	Y	NA	NA	Y	N	N	N	Y	Y	7	5	2	1	Unacceptable
Shanley et al. (2015)^a,c^	Y	Y	N	Y	CS	N	Y	NA	NA	Y	Y	Y	CS	Y	Y	9	2	2	2	Unacceptable
Slodwonik et al. (2018)^c^	Y	Y	Y	Y	50%	N	Y	CS	N	Y	Y	N	N	N	Y	8	5	0	1	Unacceptable
Smith et al. (2015)^a,c^	Y	CS	Y	CS	20%	N	N	NA	NA	Y	Y	NA	N	N	Y	5	4	3	2	Unacceptable
Struyf et al. (2014)^a,c^	Y	Y	N	Y	30%	N	N	CS	N	CS	N	N	N	N	Y	4	8	0	2	Unacceptable
Wang et al. (2001)^a,c^	Y	Y	Y	Y	CS	N	Y	CS	N	Y	N	Y	N	N	NA	7	5	1	2	Unacceptable
Wilk et al. (2011)^a,c^	Y	Y	N	Y	CS	N	Y	CS	N	Y	N	N	N	Y	Y	7	6	0	2	Unacceptable
Wright et al. (2007)^a,c^	Y	CS	N	Y	CS	N	Y	NA	NA	N	N	N	NA	N	Y	4	6	3	2	Unacceptable
Yung et al. (2007)^a,c^	Y	Y	NA	Y	0%	NA	Y	NA	NA	Y	N	NA	N	N	N	5	4	5	0	Unacceptable

*SIGN* Scottish Intercollegiate Guidelines Network, *Y* Yes, *N* No, *NA* Not applicable, *CS* Can’t say

^a^Taken from previous review (Asker et al., 2018)

^b^Randomised controlled trial with 10 items

^c^For excluded references see supplementary material

### Study characteristics

The most investigated overhead sport was baseball with 4 studies [26, 39, 40, 54] followed by handball with 3 studies [2, 4, 5]. There was one study on softball [35] and water polo [20] each. While both genders were studied in 5 studies [2, 4, 5, 20, 39], males were most likely investigated in one study due to the league affiliation [54]. There were 3 studies on baseball and softball in which the sex was not explicitly specified [26, 35, 40]. 6 studies included youth [4, 5, 26, 35, 39, 40], 2 studies adult elite [2, 54], and one study adult sub-elite [20] athletes. With respect to the study design and outcome type, 7 cohort studies investigated risk factors [4, 5, 20, 26, 35, 40, 54], whereas 2 randomised controlled trails evaluated prevention strategies [2, 39].

### Synthesis of results on risk factors

Table 3 summarises the outcomes of the 7 cohort studies on risk factors according to the PICO-framework. The most addressed risk factor was playing position with 4 studies [4, 26, 35, 40] followed by setting (match vs. training) [20, 35, 40] and gender with 3 studies [4, 5, 20], and shoulder rotational ROM with 2 studies [5, 54]. Further risk factors as history of shoulder/elbow pain [26], age [26], training experience [26], training volume [26], school grade [4], playing level [4], shoulder rotational strength [5], scapula dyskinesia [5], and joint position sense [5] were addressed in one study each.

**Table 3:** Characteristics of the 7 cohort studies on risk factors according to the PICO-framework

Author (Year)	Population	Intervention	Comparison	Outcome
Asker et al. (2020) [5]	471 female (54%) and male adolescent elite handball players from handball-profiled schools in Sweden, 15–19 years old, free of shoulder injuries at baseline	Baseline shoulder examination and questionnaire followed by weekly online monitoring of shoulder injuries over one (2014/15) or two seasons (2015/16)	Gender differences in relationship between shoulder injury incidence (dominant side) during handball play and shoulder ROM, strength, scapular dyskinesia, and joint position sense	Shoulder incidence was 0.92/1000 hrs in females and 0.71/1000 hrs in males; positive relationship between isometric shoulder external/internal rotation strength deficit and injury risk in females (hazard rate ratio: < 2.37) but not in males (< 1.02), positive relationship between scapular dyskinesia during abduction and injury risk in males (3.43) but not in females (1.53), no association with internal/external/total rotational ROM (< 1.56) and joint position sense (< 1.14) in both genders
Asker et al. (2018) [4]	471 female (54%) and male adolescent elite handball players from handball-profiled schools in Sweden, 15–19 years old, free of shoulder injuries at baseline	Baseline questionnaire followed by weekly online monitoring of shoulder injuries over one (2014/15) or two seasons (2015/16)	Gender, school grade, playing position, and playing level differences in shoulder injury prevalence	Shoulder prevalence was higher in females (< 48%, prevalence ratio: < 1.46) than in males (< 39%, 1.00) and higher in backcourt players (< 51%, < 1.58) than in other positions (< 40%, < 1.00), no differences in school grade and playing level
Hams et al. (2019a) [20]	218 female (59%) and male adult sub-elite water polo players from Australia, mean age of 19.3 and 20.6 years	Self- (2009–2013) and physiotherapist-report (2014–2016) on injury data of several body areas over up to 5 years	Body area, gender, and training/match differences in injury incidence rate, mechanism of injury, and injury burden	Shoulder incidence rate was 0.65/1000 training days, 25% (self-report) and 16% (physiotherapist-report) of all injuries being shoulder injuries with no gender differences (*p* = 0.33), proportion of shoulder injuries (16–25%) was higher than for all other body areas (11–17%) (*p* < 0.01), 67% of shoulder injuries were due to overuse and 33% due to trauma, more injuries in training (48%) than match (24%), each shoulder injury resulted in 6 days of training lost and 47 days in modified training
Matsuura et al. (2017) [26]	900 youth baseball players from Japan participating in regional summer championship, 7–11 years old	Baseline questionnaire and at one year follow-up (2012–2013)	Multivariate relationships between shoulder/elbow pain and age, playing position, length of baseball experience, training hours per week, and history of shoulder/elbow pain	Shoulder and elbow pain was evident in 18% and 35% of players; shoulder pain was only related to pitcher/catcher position, training hours per week, and history of shoulder/elbow pain (all *p* < 0.05). No relationship with age (*p* > 0.42) and length of experience (*p* > 0.52)
Oliver et al. (2019) [35]	Softball players from 100 high schools in USA, ~ 16 years old on average	Surveillance system on shoulder and elbow injury data (2005–06 to 2016–17)	Differences in injury rate, mechanism of injury, match/training occurrence, playing position distribution, injury burden, and further characteristics of shoulder and elbow injuries	Shoulder injury rate (1.14/1000 athlete-exposure) was higher than for elbow (0.41/1000), shoulder injury rate was higher in match (1.33/1000) than training (1.04/1000), 50% of shoulder injuries were due to overuse and most common diagnoses were muscle strains (31%) followed by tendinitis (24%), 17% of shoulder injuries were sustained by pitchers, 87% of all shoulder injured players returned to play within 21 days and remaining 13% did not return
Saper et al. (2018) [40]	Baseball players from 100 high schools in USA, ~ 16 years old on average	Surveillance system on shoulder and elbow injury data (2005–06 to 2016–17)	Differences in injury rate, mechanism of injury, match/training occurrence, playing position distribution, injury burden, and further characteristics of shoulder and elbow injuries	Shoulder injury rate (1.39/1000 athlete-exposure) was higher than for elbow (0.86/1000), shoulder injury rate was higher in match (1.73/1000) than training (1.20/1000), 71% of shoulder injuries were due to overuse and most common diagnoses were muscle strains (31%) followed by tendinitis (19%), 40% of shoulder injuries were sustained by pitchers, 90% of shoulder injuries were managed non-surgically, 87% of shoulder injured players returned to play within 21 days
Wilk et al. (2014) [54]	296 professional baseball pitchers from the USA participating in the major and minor league, 24.7 years old on average, free of shoulder injuries at baseline	Baseline shoulder examination and questionnaire over eight seasons (2005–2012)	Differences in shoulder ROM between dominant/non-dominant side and relationship with shoulder injuries and surgeries	17% of all pitchers suffered a shoulder injury, 7% required a surgery whereby most were labral and rotator cuff debridements (35%) followed by labral repairs (30%) and debridements (20%), pitchers showed less shoulder internal/total rotation ROM but higher external ROM in their dominant than non-dominant shoulder (all *p* < 0.01), shoulder internal/total rotation ROM deficits were not related to shoulder injury or surgery (*p* > 0.21) but shoulder external ROM deficit increased the likelihood to sustain shoulder injury (2.2 times higher) and surgery (4.0 times)

*n* Number of participants, *ROM* Range of motion

#### Playing position (4 studies)

One study in adolescent elite handball players shows a higher shoulder injury prevalence for backcourt players compared to other positions [4]. Two other studies in high school baseball and softball players show that most shoulder injuries were sustained by pitchers [35, 40]. A further study in youth regional baseball players reveals that pitcher and catcher position was a predictor for shoulder pain [26].

#### Setting (match vs. training) (3 studies)

Two studies in high school baseball and softball players show a higher shoulder injury rate during match than training [35, 40]. Contrary, a study in female and male adult sub-elite water polo players reveals a higher shoulder incidence rate during training than match [20].

#### Gender (3 studies)

Two studies in adolescent elite handball players demonstrate a higher shoulder incidence and prevalence in females than males [4, 5]. However, no gender differences of shoulder incidence rates were found in adult sub-elite water polo players [20].

#### Shoulder rotational ROM (2 studies)

A study in female and male adolescent elite handball players revealed that shoulder internal, external, and total rotational ROM was not related to new injuries [5]. Another study in professional baseball pitchers reveals that shoulder internal and total rotational ROM deficits were not related to injury or surgery. However, a positive relationship with injury and surgery was found for external rotational ROM deficit [54].

#### Further risk factors (one study each)

One study in youth regional baseball players found that the history of shoulder and elbow pain and weekly training volume were positively related to shoulder pain; however, no associations were observed for age and training experience [26]. Another study in female and male adolescent elite handball players found no differences in shoulder injury prevalence according to school grade and playing level [4]. An additional study with the same cohort observed that isometric shoulder internal and external rotation strength deficits were related to injury risk in females only, whereas scapular dyskinesia during abduction was linked to injury risk in males only. Moreover, no relationship was detected for shoulder joint position sense in both genders [5].

### Synthesis of results on prevention strategies

Table 4 summarises the outcomes of the 2 randomised controlled trails on prevention strategies according the PICO-framework. Both studies [2, 39] applied the identical block randomised study design: the teams were allocated either to an intervention group performing a 10 min prevention programme during the warm-up or to a control group performing the normal warm-up. Specifically, one study [2] investigated female and male adult elite handball players. The investigated prevention programme included exercises to improve internal rotation ROM, shoulder external rotation and scapular strength, kinetic chain, and thoracic mobility. The programme was performed 3 times per week over 7 months. Contrary, the other study [39] addressed female and male youth baseball players playing at a regional level. In that study, the evaluated prevention programme consisted of stretching exercises to enhance shoulder, elbow, and hip ROM, dynamic mobility exercises to enhance scapular and thoracic function, and lower extremity exercises to enhance balance performance. The programme was performed at least once per week over 12 months.

**Table 4:** Characteristics of the 2 randomised controlled trails on prevention strategies according to the PICO-framework

Study (Year)	Population	Intervention	Comparison	Outcome
Andersson et al. (2017) [2]	660 female (49%) and male adult elite handball players from 45 teams participating in two highest leagues in Norway, ~ 22 years old on average, participating irrespective of shoulder injury status at baseline	Teams were block randomised into intervention (*n* = 22 teams, 331 players) and control group (*n* = 23 teams, 329 players); intervention group performed the Oslo Sports Trauma Research Centre Shoulder Injury Prevention Program (10 min exercises to improve internal rotation ROM, shoulder external rotation/scapular strength, kinetic chain, and thoracic mobility) 3 times per week during warm-up over 7 months (2014/15), control group performed normal warm-up; baseline questionnaire followed by monthly online monitoring of shoulder injuries	Group differences between prevalence of shoulder problems and substantial shoulder problems (moderate/severe reductions in training or inability to participate therein) in dominant arm	Prevalence of shoulder problems/substantial shoulder problems was 17%/5% in intervention and 23%/8% in control group during observation period, intervention group had 28% lower risk to sustain shoulder problems (odds ratio: 0.72, *p* = 0.04) than control group, no differences between groups for substantial shoulder problems (odds ratio: 0.78, *p* = 0.23)
Sakata et al. (2019) [39]	219 female (< 1%) and male youth baseball players from 16 teams participating in regional league in Japan, 9–11 years old, participating irrespective of shoulder injury status at baseline	Teams were block randomised into intervention (*n* = 8 teams, 117 players) and control group (*n* = 8 teams, 120 players); intervention group performed the modified Yokohama Baseball-9 Throwing Injury Prevention Program (10 min stretching exercises to improve shoulder/elbow/hip ROM, dynamic mobility exercises to improve scapular/thoracic function, and lower extremity exercises to improve balance) at least once per week during warm-up over 12 months (2015/16), control group performed normal warm-up; baseline questionnaire followed by clinical/ultrasonographic shoulder assessment every 4 months and ball throwing speed pre/post intervention	Group differences between incidence of shoulder and/or elbow injuries; ball throwing speed as performance measure; and differences in defined risk factors as shoulder/elbow/hip ROM, thoracic kyphosis angle, and modified Star Excursion Balance Test performance	Incidence of pooled shoulder and/or elbow injuries was lower (hazard ratio: 1.94, *p* = 0.010) in intervention (1.7/1000 athlete exposures) than control group (3.1/1000), no differences for isolated shoulder (hazard ratio: 2.08, *p* = 0.076) and elbow injuries (hazard ratio: 1.79, *p* = 0.052); ball throwing speed increased more on average (*p* = 0.010) in intervention (+ 6.4 km/h) than control group (+ 4.1 km/h); intervention group showed also improved shoulder horizontal adduction ROM deficit in dominant side, hip internal rotational ROM in non-dominant side, and thoracic kyphosis angle (*p* < 0.03)

*n* Number of participants, *ROM* Range of motion

#### Effectiveness of prevention programmes

The first study [2] shows that the prevention programme decreased the risk to sustain shoulder problems by 28%. However, the programme was not effective to decrease shoulder problems that were moderate and severe. The second study [39] reveals that the prevention programme reduced the pooled shoulder and elbow injuries. While the programme was not effective for isolated shoulder and elbow injuries, it also improved the ball throwing speed as a performance measure and the shoulder horizontal adduction ROM deficit in dominant side, hip internal rotation ROM in non-dominant side, and thoracic kyphosis angle as some of the additionally investigated potential underlying risk factors.

### Best-evidence synthesis

Table 5 summarises the outcomes of the best-evidence synthesis. There was no risk factor or prevention strategy for which strong evidence could be identified. However, moderate evidence was found for two non-modifiable (playing position and gender) and three modifiable factors (shoulder rotational strength, scapular dyskinesia, and shoulder prevention programme) that were all associated with the risk to sustain a shoulder injury. All further risk factors had moderate and no association with risk (shoulder rotational ROM and joint position sense) or limited (history of shoulder/elbow pain, age, training experience, training volume, school grade, and playing level), and conflicting evidence (setting).

**Table 5:** Best-evidence synthesis of risk factors and prevention strategies

Study (Year)	Risk factor / prevention strategy	Association with risk	Study quality	Rating
Asker et al. (2018) [4]	Playing position	↑	Acceptable	Moderate evidence
Matsuura et al. (2017) [26]		↑	Acceptable	
Oliver et al. 2019 [35]		↑	Acceptable	
Saper et al. (2018) [40]		↑	Acceptable	
Hams et al. (2019a) [20]	Setting (match vs. training)	↓	Acceptable	Conflicting evidence
Oliver et al. (2019) [35]		↑	Acceptable	
Saper et al. (2018) [40]		↑	Acceptable	
Asker et al. (2020) [5]	Gender	↑	High quality	Moderate evidence
Asker et al. (2018) [4]		↑	Acceptable	
Hams et al. (2019a) [20]		→	Acceptable	
Asker et al. (2020) [5]	Shoulder rotational ROM	→^a^	High quality	Moderate evidence
Wilk et al. (2014) [54]		→^b^	Acceptable	
Matsuura et al. (2017) [26]	History of shoulder/elbow pain	↑	Acceptable	Limited evidence
Matsuura et al. (2017) [26]	Age	→	Acceptable	Limited evidence
Matsuura et al. (2017) [26]	Training experience	→	Acceptable	Limited evidence
Matsuura et al. (2017) [26]	Training volume	↑	Acceptable	Limited evidence
Asker et al. (2018) [4]	School grade	→	Acceptable	Limited evidence
Asker et al. (2018) [4]	Playing level	→	Acceptable	Limited evidence
Asker et al. (2020) [5]	Shoulder rotational strength	↓^c^	High quality	Moderate evidence
Asker et al. (2020) [5]	Scapular dyskinesia	↑^d^	High quality	Moderate evidence
Asker et al. (2020) [5]	Joint position sense	→	High quality	Moderate evidence
Andersson et al. (2017) [2]	Prevention programme	↓	Acceptable	Moderate evidence
Sakata et al. (2019) [39]		↓	Acceptable	

*ROM* Range of motion

↑ Positive association; ↓ Negative association; → No association

^a^For internal, external, and total rotational ROM

^b^With exception of external rotational ROM deficit, where a positive association is evident

^c^For females only

^d^For males only

## Discussion

Our systematic review found moderate evidence for five factors being associated with the risk to sustain a shoulder injury in overhead sports (playing position, gender, shoulder rotational strength, scapular dyskinesia, shoulder prevention programme), which is in contrast to a previous and methodological similar review showing limited and conflicting evidence in 2018 [3]. While the previous review could include only three studies with at least an acceptable quality [2, 26, 54], we were able to add additional 6 studies [4, 5, 20, 35, 39, 40] to the best-evidence synthesis (Table 5) explaining the discrepancies. However, our outcomes (Tables 3 and 4) also reveal a lack of methodological acceptable research for overhead sports except for baseball and handball as well as in adult athletes. Additionally, little knowledge exists for numerous clinically established risk factors [8, 11, 13, 18, 19]. Moreover, there exist only two randomised controlled trails evaluating the effectiveness of shoulder prevention programmes [2, 39]. Overall, the knowledge on risk factors and prevention strategies for shoulder injuries in overhead sports based on acceptable methodological studies (Tables 3, 4 and 5) has increased during the last 3 years, legitimising an update as conducted here, but is clearly beyond that existing for other severe sports injuries such as anterior cruciate ligament injuries [52].

Our study shows moderate evidence for the playing position as a non-modifiable risk factor to sustain a shoulder injury in overhead sports (Tables 3 and 5). One explanation is that the mechanical loading of the shoulder joint differs according to the position-specific playing demands in overhead sports. In fact, handball backcourt players throw more often at high-speed on the goal and perform more passes than other playing positions [22]. Also, they are more often involved in tactical situations placing the shoulder in vulnerable positions, for example, when stopped by opponents during breakthroughs [22]. Similarly, baseball/softball pitchers, and also catchers, perform more high-speed throws than the other positions [28, 36]. With respect to high-speed throws, it is known that they induce high-forces to the shoulder joint [53], which can lead to an accumulation of microtrauma and increase the injury and overuse risk [1, 33] for certain playing positions as observed here (Tables 3 and 5). However, all of the 4 included studies were conducted in youth handball and baseball/softball athletes competing at high playing levels [4, 26, 35, 40]. Therefore, it remains unclear, whether a young age and high playing level are interacting risk factors here [26] for which we found however limited evidence in isolation (Table 5). To clarify this, more research is needed.

Also, moderate evidence was detected for the gender as a further non-modifiable shoulder injury risk factor (Tables 3 and 5). While the underlying mechanisms remain unknown, different throwing kinematics may be one factor for the higher injury risk in females [45, 48], but there is no study showing a causal relationship yet [4]. Interestingly, the higher injury risk for females is also known from other severe sport injuries, in particular, to the anterior cruciate ligament [51]. Thereby, a higher laxity is considered as one explanatory factor [21]. From a clinical point of view, a high laxity was also expected as a risk factor for shoulder injuries in overhead sports [18], but we were unable to detected any evidence therefore (Tables 3 and 5). Again, it is also worth mentioning here that the higher risk for females was shown by 2 studies in youth elite handball athletes [4, 5], whereby the third study revealed no gender-differences at an adult sub-elite level [20]. These observations may support our previous assumption that a young age and high playing level are interacting risk factors also here, requiring further investigations.

We revealed moderate evidence for the shoulder rotational strength and scapular dyskinesia being associated with the shoulder injury risk (Tables 3 and 5). Both modifiable risk factors were investigated in one study [5], in which gender-specific relationships were detected in youth elite handball players: While isometric shoulder internal and external rotational strength deficits were associated with injury risk in females, scapular dyskinesia during abduction was related to injury risk in males. It has been speculated that these observations are also related to differences in throwing kinematics, because females use a more rotational strength demanding technique compared to males [5]. Additionally, it was pointed out that scapular dyskinesia during abduction is a clinical rational risk factor due to its close relation to the throwing technique in handball [5]. However, a consensus statement on the clinical implications concluded that scapular dyskinesis is evident in many shoulder injuries, particularly in shoulder impingement symptoms, whereby its exact role for creating or exacerbating shoulder dysfunction are not fully understood [23]. The causal factors may be related to muscular shoulder weakness, fatigue, or imbalance due to their well-known negative associations to the performance and neuromuscular control of peri-scapular muscles [12, 14].

Finally, our review shows moderate evidence that performing shoulder prevention programmes reduce the injury risk in overhead sports (Tables 4 and 5). The two included studies were conducted in female and male handball and baseball players competing at sub-elite to elite levels. Although both programmes were effective to reduce shoulder as well as pooled shoulder and/or elbow injuries, they failed to decrease the risk for substantial and isolated shoulder injuries [2, 39]. Since no negative effects are known yet, the shoulder prevention programmes can be recommended to be implemented in the training process of overhead athletes with the drawback that the exact mechanistic functioning remain widely unknown [44]. Generally, injury prevention programmes consist of several exercises performed for approximately 10 min during the warm-up. It is assumed that these exercises positively address several modifiable underlying risk factors, which finally reduce the injury risk – ideally increasing the physical performance too [44]. With respect to shoulder injuries, there is only one noteworthy study that has investigated the effectiveness of a prevention programme on all injury risk, potential underlying risk factors, and performance [39]. However, to date, nothing is known concerning the long-term effects of shoulder prevention programmes [19], effectiveness of individualised shoulder prevention programmes based on screening test results [6], impact of single exercises of an entire prevention programme, or their interdependent relationships [19] as well as optimal implementation and compliance strategies [34, 37, 43] (Tables 3, 4 and 5).

## Conclusion

There is moderate evidence for two non-modifiable (playing position, gender) and three modifiable factors (shoulder rotational strength, scapular dyskinesia, shoulder prevention programme) being associated with the shoulder injury risk in overhead sports. From a practical point of view, these factors can be used as a framework to design injury screening tests and prevention strategies that should then be adapted for each overhead sport and subpopulation. However, more research is needed to evaluate further risk factors and shoulder prevention strategies.

## Supplementary Information

**Additional file 1.**

## Data Availability

The datasets used and analysed during the current study are available from the corresponding author on reasonable request.

## Abbreviations

PRISMA: Preferred Reporting Items for Systematic Reviews and Meta-analyses
ROM: Range of motion
SIGN: Scottish Intercollegiate Guidelines Network
