# Supplementary material for: Risk factors and prevention strategies for shoulder injuries in overhead sports: an updated systematic review
Source: J Exp Orthop. 2022 Aug 16;9:78. doi: 10.1186/s40634-022-00493-9 (PMC9378805; doi:10.1186/s40634-022-00493-9)
Supplement: Supplementary file 1 — Additional file 1. [file 40634_2022_493_MOESM1_ESM.docx]

Excluded studies based on risk of bias assessment form our review.

Achenbach L, Laver L, Walter SS, Zeman F, Kuhr M, Krutsch W (2020) Decreased external rotation strength is a risk factor for overuse shoulder injury in youth elite handball athletes. Knee Surg Sports Traumatol Arthrosc 28:1202-1211.

Berardi M, Lenabat P, Fabre T, Ballas R (2020) Beach tennis injuries: a cross-sectional survey of 206 elite and recreational players. Phys Sportsmed 48:173-178.

Cunado-Gonzalez A, Martin-Pintado-Zugasti A, Rodriguez-Fernandez AL (2019) Prevalence and factors associated with injuries in elite spanish volleyball. J Sport Rehabil 28:796-802.

Dakic JG, Smith B, Gosling CM, Perraton LG (2018) Musculoskeletal injury profiles in professional Women's Tennis Association players. Br J Sports Med 52:723-729.

Dutton M, Tam N, Brown JC, Gray J (2019) The cricketer's shoulder: not a classic throwing shoulder. Phys Ther Sport 37:120-127.

Erickson BJ, Chalmers PN, Zajac J, Sgroi T, Eno JJ, Altchek DW, et al. (2019) Do professional baseball players with a higher valgus carrying angle have an increased risk of shoulder and elbow injuries? Orthop J Sports Med 7:2325967119866734.

Fares MY, Salhab HA, Khachfe HH, Kane L, Fares Y, Fares J, et al. (2020) Upper limb injuries in Major League Baseball. Phys Ther Sport 41:49-54.

Hams A, Evans K, Adams R, Waddington G, Witchalls J (2019b) Reduced shoulder strength and change in range of motion are risk factors for shoulder injury in water polo players. Phys Ther Sport 40:231-237.

Hansen C, Sanz Lopez F, Whiteley R, Wilhelm A, Popovic N, Ahmed HA, et al. (2019) [A video-based analysis to classify shoulder injuries during the Handball World Championships 2015]. Sportverletz Sportschaden 33:30-35.

Kim Y, Lee JM, Wellsandt E, Rosen AB (2020) Comparison of shoulder range of motion, strength, and upper quarter dynamic balance between NCAA division I overhead athletes with and without a history of shoulder injury. Phys Ther Sport 42:53-60.

Luig P, Krutsch W, Henke T, Klein C, Bloch H, Platen P, et al. (2020) Contact - but not foul play - dominates injury mechanisms in men's professional handball: a video match analysis of 580 injuries. Br J Sports Med 54:984-990.

Marchena-Rodriguez A, Gijon-Nogueron G, Cabello-Manrique D, Ortega-Avila AB (2020) Incidence of injuries among amateur badminton players: a cross-sectional study. Medicine (Baltimore) 99:e19785.

Olivier B, Gray J (2018) Musculoskeletal predictors of non-contact injury in cricketers - few and far between? A longitudinal cohort study. Phys Ther Sport 34:208-215.

Rugg CM, Wang D, Mayer EN, Sulzicki PL, Vail J, Hame SL (2019) The impact of prior upper-extremity surgery on orthopedic injury and surgery in collegiate athletes. J Shoulder Elbow Surg 28:1371-1377.

Salzer S, Heansel R, Rey J, Maltry L, Holzgreve F, Lampe J, et al. (2020) Injury occurrence in male handball players and its impact on physical constitution. Int J Sports Med 41:328-338.

Sekiguchi T, Hagiwara Y, Yabe Y, Tsuchiya M, Itaya N, Yoshida S, et al. (2018) Playing video games for more than 3 hours a day is associated with shoulder and elbow pain in elite young male baseball players. J Shoulder Elbow Surg 27:1629-1635.

Sekiguchi T, Hagiwara Y, Yabe Y, Tsuchiya M, Itaya N, Yoshida S, et al. (2020) Restriction in the hip internal rotation of the stride leg is associated with elbow and shoulder pain in elite young baseball players. J Shoulder Elbow Surg 29:139-145.

Slodownik R, Ogonowska-Slodownik A, Morgulec-Adamowicz N (2018) Functional Movement Screen and history of injury in the assessment of potential risk of injury among team handball players. J Sports Med Phys Fitness 58:1281-1286.

Takagishi K, Matsuura T, Masatomi T, Chosa E, Tajika T, Iwama T, et al. (2019) Shoulder and elbow pain in junior high school baseball players: results of a nationwide survey. J Orthop Sci 24:708-714.

Excluded studies based on the risk of bias assessment from the previous review (Asker et al., 2018).

Agel J, Palmieri-Smith RM, Dick R, Wojtys EM, Marshall SW (2007) Descriptive epidemiology of collegiate women’s volleyball injuries: National Collegiate Athletic Association Injury Surveillance System, 1988-1989 through 2003-2004. J Athl Train 42:295-302.

Bere T, Kruczynski J, Veintimilla N, Hamu Y, Bahr R (2015) Injury risk is low among world-class volleyball players: 4-year data from the FIVB Injury Surveillance System. Br J Sports Med 49:1132-1137.

Bonza JE, Fields SK, Yard EE, Comstock RD (2009) Shoulder injuries among United States high school athletes during the 2005-2006 and 2006-2007 school years. J Athl Train 44:76-83.

Byram IR, Bushnell BD, Dugger K, Charron K, Harrell FE, Noonan TJ (2010) Preseason shoulder strength measurements in professional baseball pitchers: identifying players at risk for injury. Am J Sports Med 38:1375-1382.

Clarsen B, Bahr R, Andersson SH, Munk R, Myklebust G (2014) Reduced glenohumeral rotation, external rotation weakness and scapular dyskinesis are risk factors for shoulder injuries among elite male handball players: a prospective cohort study. Br J Sports Med 48:1327-1333.

Dick R, Romani WA, Agel J, Case JG, Marshall SW (2007a) Descriptive epidemiology of collegiate men’s lacrosse injuries: National Collegiate Athletic Association Injury Surveillance System, 1988-1989 through 2003-2004. J Athl Train 42:255-261.

Dick R, Sauers EL, Agel J, Keuter G, Marshall SW, McCarty K, et al. (2007b) Descriptive epidemiology of collegiate men’s baseball injuries: National Collegiate Athletic Association Injury Surveillance System, 1988-1989 through 2003-2004. J Athl Train 42:183-193.

Forthomme B, Wieczorek V, Frisch A, Crielaard JM, Croisier JL (2013) Shoulder pain among high-level volleyball players and preseason features. Med Sci Sports Exerc 45:1852-1860.

Gardner EC, Chan WW, Sutton KM, Blaine TA (2016) Shoulder Injuries in men’s collegiate lacrosse, 2004-2009. Am J Sports Med 44:2675-2681.

Giroto N, Hespanhol Junior LC, Gomes MR, Lopes AD (2017) Incidence and risk factors of injuries in Brazilian elite handball players: A prospective cohort study. Scand J Med Sci Sports 27:195-202.

Gregory PL, Batt ME, Wallace WA (2002) Comparing injuries of spin bowling with fast bowling in young cricketers. Clin J Sport Med 12:107-112.

Hibberd EE, Kerr ZY, Roos KG, Djoko A, Dompier TP (2016) Epidemiology of acromioclavicular joint sprains in 25 National Collegiate Athletic Association sports: 2009-2010 to 2014-2015 academic years. Am J Sports Med 44:2667-2674.

Hinton RY, Lincoln AE, Almquist JL, Douoguih WA, Sharma KM (2005) Epidemiology of lacrosse injuries in high school-aged girls and boys: a 3-year prospective study. Am J Sports Med 33:1305-1314.

Lyman S, Fleisig GS, Andrews JR, Osinski ED (2002) Effect of pitch type, pitch count, and pitching mechanics on risk of elbow and shoulder pain in youth baseball pitchers. Am J Sports Med 30:463-8.

Lyman S, Fleisig GS, Waterbor JW, Funkhouseer EM , Pulley L, Andrews JR, et al. (2001) Longitudinal study of elbow and shoulder pain in youth baseball pitchers. Med Sci Sports Exerc 33:1803-1810.

Marshall SW, Hamstra-Wright KL, Dick R, Grove KA, Agel J (2007) Descriptive epidemiology of collegiate women’s softball injuries: National Collegiate Athletic Association Injury Surveillance System, 1988-1989 through 2003-2004. J Athl Train 42:286-294.

Møller M, Nielsen RO, Attermann J, Wedderkopp N, Lind M, Sørensen H, et al. (2017) Handball load and shoulder injury rate: a 31-week cohort study of 679 elite youth handball players. Br J Sports Med 51:231-237.

Noonan TJ, Thigpen CA, Bailey LB, Wyland DJ, Kissenberth M, Hawkins RJ, et al. (2016) Humeral torsion as a risk factor for shoulder and elbow injury in professional baseball pitchers. Am J Sports Med 44:2214-2219.

Owens BD, Agel J, Mountcastle SB, Cameron KL, Nelson BJ (2009) Incidence of glenohumeral instability in collegiate athletics. Am J Sports Med 37:1750-1754.

Polster JM, Bullen J, Obuchowski NA, Bryan JA, Soloff L, Schickendantz MS (2013) Relationship between humeral torsion and injury in professional baseball pitchers. Am J Sports Med 41:2015-2021.

Polster JM, Lynch TS, Bullen JA, Soloff L, Ilaslan H, Subhas N, et al. (2016) Throwing-related injuries of the subscapularis in professional baseball players. Skeletal Radiol 45:41-47.

Ranson C, Gregory PL. (2008) Shoulder injury in professional cricketers. Phys Ther Sport 9:34-39.

Reeser JC, Gregory A, Berg RL, Comstock RD (2015) A comparison of women’s collegiate and girls’ high school volleyball injury data collected prospectively over a 4-year period. Sports Health 7:504-510.

Robinson TW, Corlette J, Collins CL, Comstock RD (2014) Shoulder injuries among US high school athletes, 2005/2006-2011/2012. Pediatrics 133:272-279.

Sallis RE, Jones K, Sunshine S, Smith G, Simon L (2001) Comparing sports injuries in men and women. Int J Sports Med 22:420-423.

Seil R, Rupp S, Tempelhof S, Kohn D (1998) Sports injuries in team handball. A one-year prospective study of sixteen men’s senior teams of a superior nonprofessional level. Am J Sports Med 26:681-687.

Sell K, Hainline B, Yorio M, Kovacs M (2014) Injury trend analysis from the US Open Tennis Championships between 1994 and 2009. Br J Sports Med 48:546-551.

Shanley E, Rauh MJ, Michener LA, Ellenbecker TS (2011) Incidence of injuries in high school softball and baseball players. J Athl Train 46:648-654.

Shanley E, Kissenberth MJ, Thigpen CA, Bailey LB, Hawkins RJ, Michener LA, et al. (2015) Preseason shoulder range of motion screening as a predictor of injury among youth and adolescent baseball pitchers. J Shoulder Elbow Surg 24:1005-1013.

Smith MV, Davis R, Brophy RH, Prather H, Garbutt J, Wright RW (2015) Prospective player-reported injuries in female youth fast-pitch softball players. Sports Health 7:497-503.

Struyf F, Nijs J, Meeus M, Roussel NA, Mottram S, Truijen S, et al. (2014) Does scapular positioning predict shoulder pain in recreational overhead athletes? Int J Sports Med 35:75-82.

Wang HK, Cochrane T (2001) A descriptive epidemiological study of shoulder injury in top level English male volleyball players. Int J Sports Med 22:159-163.

Wilk KE, Macrina LC, Fleisig GS, Porterfield R, Simpson 2^nd^ CD, Harker P, et al. (2011) Correlation of glenohumeral internal rotation deficit and total rotational motion to shoulder injuries in professional baseball pitchers. Am J Sports Med 39:329-335.

Wright RW, Steger-May K, Klein SE (2007) Radiographic findings in the shoulder and elbow of Major League Baseball pitchers. Am J Sports Med 35:1839-1843.

Yung PS, Chan RH, Wong FC, Cheuk PW, Fong DT (2007) Epidemiology of injuries in Hong Kong elite badminton athletes. Res Sports Med 15:133-146.
